# Biomaterial-Free Three-Dimensional Bioprinting of Cardiac Tissue using Human Induced Pluripotent Stem Cell Derived Cardiomyocytes

**DOI:** 10.1038/s41598-017-05018-4

**Published:** 2017-07-04

**Authors:** Chin Siang Ong, Takuma Fukunishi, Huaitao Zhang, Chen Yu Huang, Andrew Nashed, Adriana Blazeski, Deborah DiSilvestre, Luca Vricella, John Conte, Leslie Tung, Gordon F. Tomaselli, Narutoshi Hibino

**Affiliations:** 1 Division of Cardiac Surgery, Johns Hopkins Hospital, Baltimore, Maryland USA; 2 Division of Cardiology, Johns Hopkins Hospital, Baltimore, Maryland USA; 3 Department of Biomedical Engineering, Johns Hopkins University, Baltimore, MD USA

## Abstract

We have developed a novel method to deliver stem cells using 3D bioprinted cardiac patches, free of biomaterials. Human induced pluripotent stem cell-derived cardiomyocytes (hiPSC-CMs), fibroblasts (FB) and endothelial cells (EC) were aggregated to create mixed cell spheroids. Cardiac patches were created from spheroids (CM:FB:EC = 70:15:15, 70:0:30, 45:40:15) using a 3D bioprinter. Cardiac patches were analyzed with light and video microscopy, immunohistochemistry, immunofluorescence, cell viability assays and optical electrical mapping. Cardiac tissue patches of all cell ratios beat spontaneously after 3D bioprinting. Patches exhibited ventricular-like action potential waveforms and uniform electrical conduction throughout the patch. Conduction velocities were higher and action potential durations were significantly longer in patches containing a lower percentage of FBs. Immunohistochemistry revealed staining for CM, FB and EC markers, with rudimentary CD31+ blood vessel formation. Immunofluorescence revealed the presence of Cx43, the main cardiac gap junction protein, localized to cell-cell borders. *In vivo* implantation suggests vascularization of 3D bioprinted cardiac patches with engraftment into native rat myocardium. This constitutes a significant step towards a new generation of stem cell-based treatment for heart failure.

## Introduction

Heart failure is common, debilitating and ultimately lethal malady which consumes billions of dollars and thousands of lives each year^1^. Current treatments for heart failure^2^ alleviate symptoms and modestly prolong life, but are palliative as they do not address the fundamental mechanism of the loss of functional cardiac tissue. Cellular therapy aimed at improving cardiac function and regenerating new myocardium, has been extensively investigated for cardiac repair^3^. Despite numerous preclinical and clinical studies^4–9^ performed to assess the ability of various stem cell populations to improve cardiac function and reduce infarct size, many important issues remain unresolved, and no cell therapy to date has been demonstrated conclusively to be effective. There are significant limitations to cell therapy in terms of cell retention, survival of the engrafted cells, cell differentiation, and integration of transplanted cells with host tissue^10^.

In order to overcome the current issues in cell-based treatment of heart failure, the key factors are: (1) Effective delivery of an abundant number of myocytes or progenitor cells, (2) improvement of retention and engraftment of delivered cells with sufficient nutritional supply, (3) Development of biomaterial that can directly effect the contraction of cardiac tissue. Recent advances have applied 3D printing technologies to biocompatible materials, cells and supporting components with great promise for artificial organ printing and regenerative medicine applications^11^. 3D bioprinting of myocardial tissue has utilized biomaterials^12, 13^, such as polycaprolactone (PCL)^14^, sodium alginate^15^, 3D printed gelatin/methylbenzene sulfonate (MBS) extracellular matrix (ECM) scaffolds^16^ or decellularized ECM to generate platforms for extrusion-based printing^17^. The use of biomaterials face challenges, such as immunogenicity, host inflammatory responses, fibrous tissue formation, biomaterial degradation and toxicity of degradation products, that affect the long term function of the engineered tissue construct^18^.

**Figure 1:**
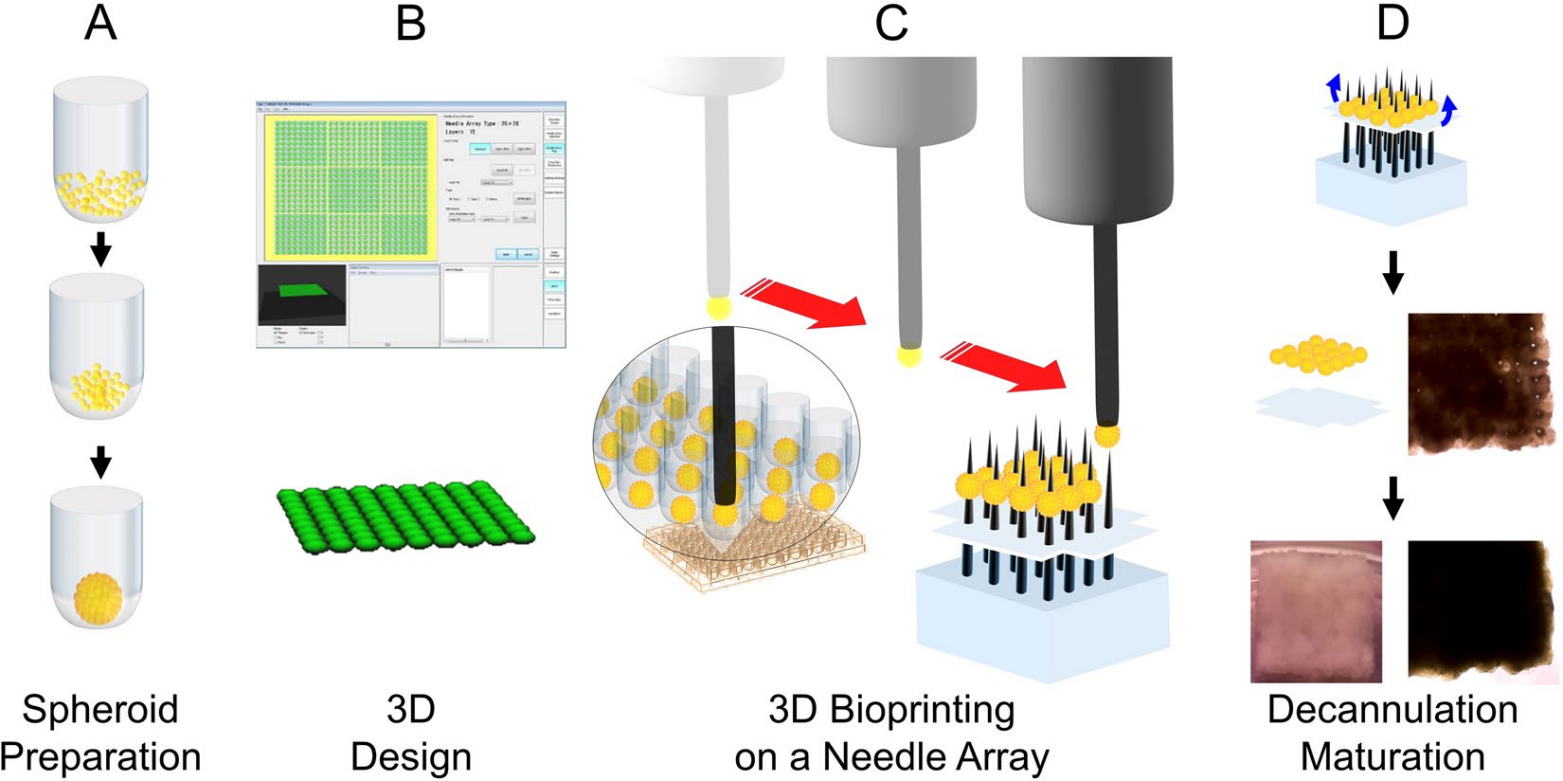
Schematic overview of biomaterial-free cardiac 3D bioprinting process. (**A**) Cells (CMs, FBs, ECs) are aggregated in ultra-low attachment 96-well plates to form cardiospheres. (**B**) The desired 3D structure to be bioprinted is designed using computer software. (**C**) The 3D bioprinter picks up individual cardiospheres using vacuum suction and loads them onto a needle array. (**D**) Cardiospheres are allowed to fuse. The 3D bioprinted cardiac tissue is then removed from the needle array, and cultured further to allow the needle holes to be filled in by surrounding tissue.

We have developed a method to create 3D bioprinted cardiac tissue without the use of biomaterials^19^ (Fig. 1), by assembling multicellular cardiospheres consisting of human induced pluripotent stem cell derived cardiomyocytes (hiPSC-CMs), human adult ventricular cardiac fibroblasts (FBs) and human umbilical vein endothelial cells (ECs) using a 3D bioprinter. We hypothesize that novel biomaterial-free 3D printed cardiac patches will exhibit mechanical integrity with electrical integration of component cardiospheres.

## Results

### hiPSC-CMs and cardiosphere formation

**Figure 2:**
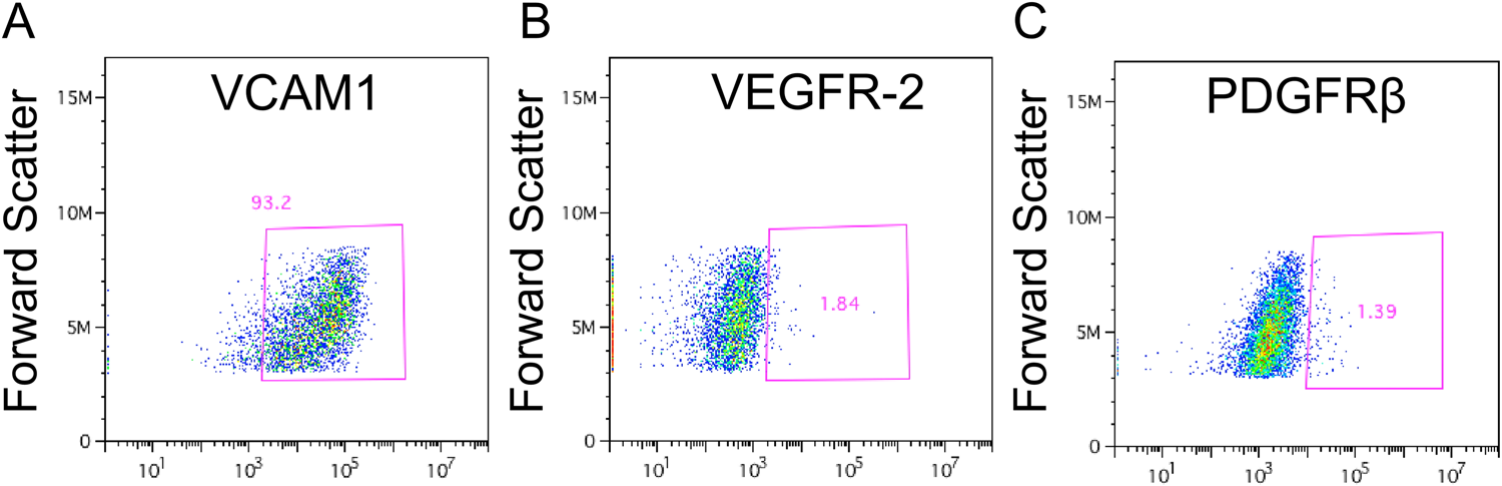
Cell surface markers expressed by hiPSC-CMs used to generate cardiospheres. (**A**–**C**) Flow cytometry analysis using VCAM1 (**A**), VEGFR-2 (**B**), PDGFRβ (**C**) surface markers.

We generated hiPSC-CMs that were enriched for CM cell surface markers, as demonstrated by flow cytometry performed on hiPSC-CMs 14 days post differentiation (Fig. 2A–C). VCAM1, a cardiomyocyte marker^20^, was predominantly expressed (93.2%). Of the 6.8% remaining cells, 1.8% and 1.4% were positive for the vascular and fibroblast markers VEGFR-2 and PDGFRβ. Further hiPSC-CM quality control measures (spontaneous beating phenotype, electrical connections by voltage optical mapping) are described in Methods.

**Figure 3:**
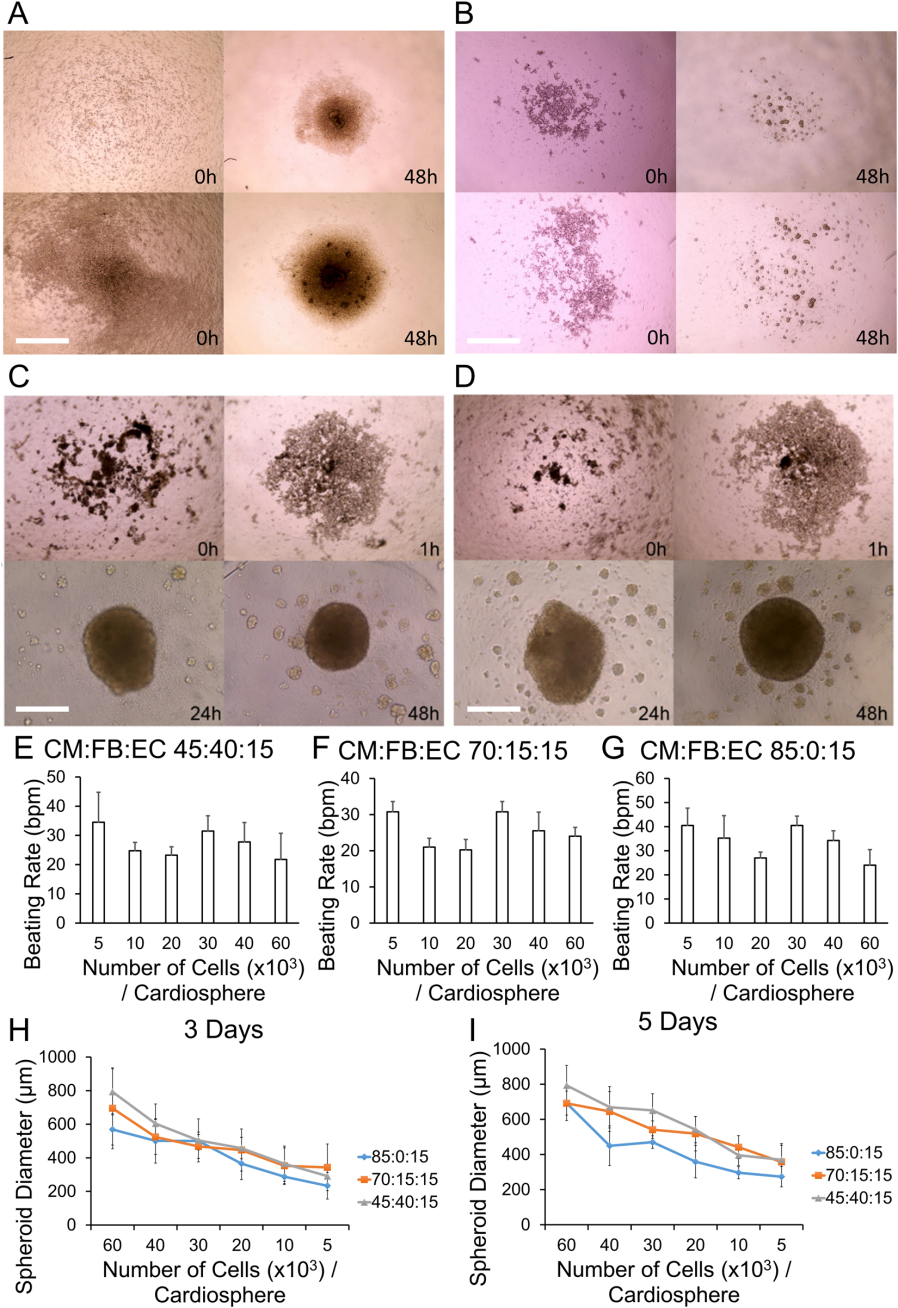
Cell type, number and ratio determine cardiosphere integrity. (**A**,**B**) hiPSCs (100%) (**A**) and hiPSC-CMs (100%) (**B**) do not form spheroids after 48 hours in ultra-low attachment 96-well plates (Top 10,000 cells/well. Bottom 40,000 cells/well). (**C**,**D**) hiPSC-CM:FB:EC ratio 70:15:15 (**C**) and 45:45:10 (**D**): Cardiospheres form in 24 hours and start beating in 48 hours. Scale bar panels A–D: 400 µm. (**E**–**G**) Beating rate of cardiospheres composed of hiPSC-CM:FB:EC ratio of 45:40:15 (**E**), 70:15:15 (**F**) and 85:0:15 (**G**) at various cell numbers per cardiosphere, in beats per minute (bpm). (**H**,**I**) Mean diameter (µm) of cardiospheres of various cell ratios (hiPSC-CM:FB:EC) and various cell numbers, after 3 days (**H**) and 5 days (**I**) of culture.

In order to create cardiospheres that were amenable to 3D bioprinting, we had to optimize the cell types, cell ratios and total cell numbers required for aggregation in each well. A fixed endothelial cell component of 15% was chosen based on a previous report demonstrating prevascularization of cardiac patches using endothelial cells^21^. Cardiospheres did not form when only hiPSCs or hiPSC-CMs were used; (Figs S2 and 3A,B). In the case of hiPSCs (Fig. 3A), a loose, soft, gel-like cell aggregate, that is easily disrupted, formed within 48 hours. In the presence of FBs and ECs, hiPSC-CMs formed cardiospheres in 24 hours and these cardiospheres started beating spontaneously by 48 hours (Fig. 3C,D). The spheroid beating rate was 28.7 ± 7.9 beats per minute (mean ± SD) (Fig. 3E–G). We then created cardiospheres of different cell ratios (CM:FB:EC 85:0:15, 70:15:15, 45:40:15) and various cell densities (5, 10, 20, 30, 40, 60 thousand cells/cardiosphere), and measured their diameters after 3 days of culture (Fig. 3H) and 5 days of culture (Fig. 3I), to determine the mean cardiosphere diameters respectively.

**Figure 4:**
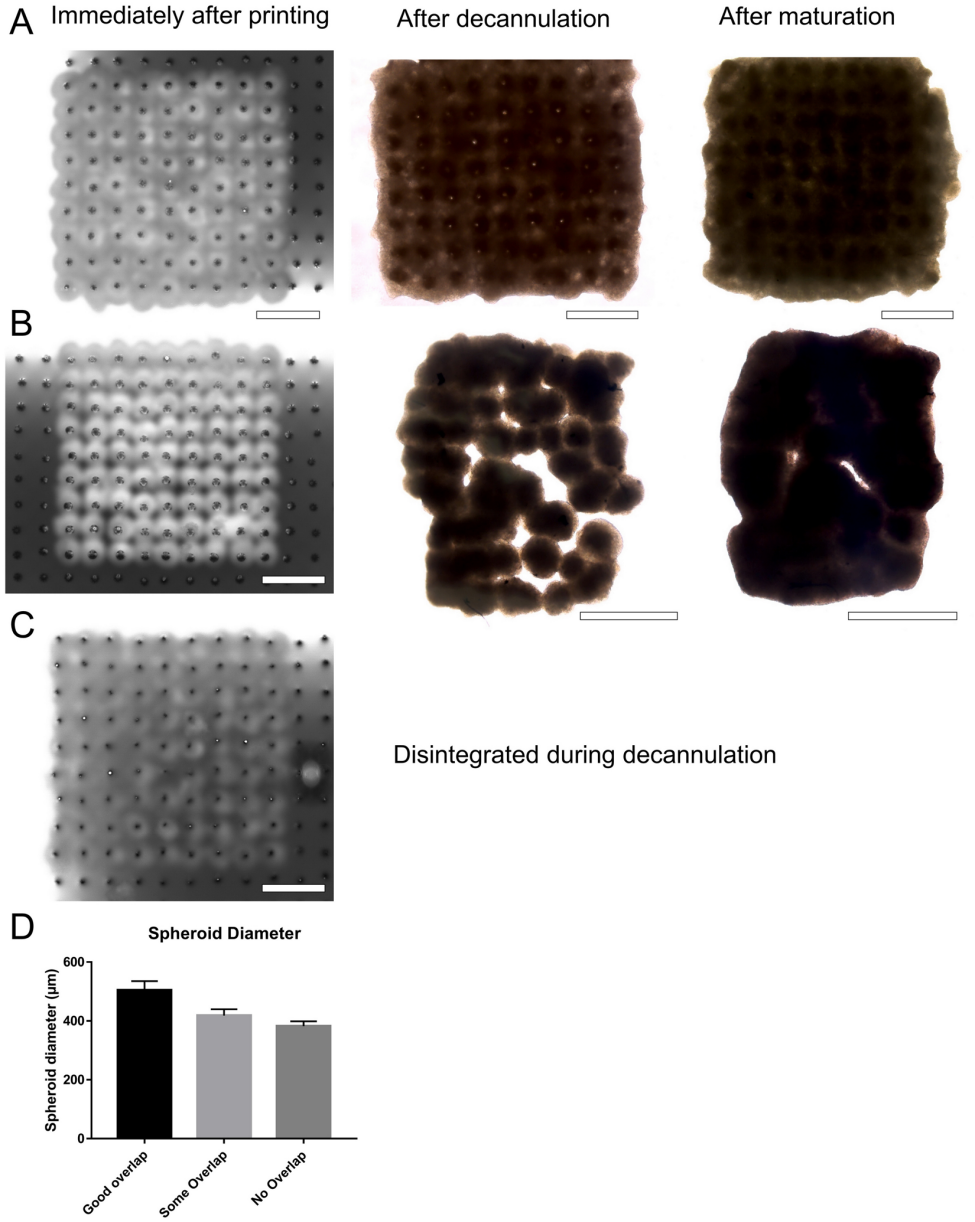
Cardiosphere dimensions and the degree of cardiosphere overlap determines the structural properties of 3D bioprinted cardiac patches. (**A**–**C**) Representative images of 3D bioprinted cardiac patches (CM:FB:EC 70:15:15) with overlap at least 50 µm (**A**), some overlap, 1 to 50 µm (**B**) and no overlap (**C**), immediately after printing, after decannulation and after maturation. Scale bar: 1000 µm. (**D**) Cardiosphere diameters immediately before printing for 3D bioprinted cardiac patches in Panels A (504.6 ± 30.6 µm) (n = 81), B (413.1 ± 21.6 µm) (n = 81), C (382.4 ± 16.1 µm) (n = 65) respectively.

Sufficient overlap of cardiospheres was needed for mechanical integrity of the 3D bioprinted cardiac patches (Fig. 4A,B). When the borders of the cardiospheres did not overlap sufficiently, the subsequent fusion of cardiospheres was poor and, upon decannulation from the needle array the patches tended to disintegrate. Interestingly, even though there were holes and partial disintegration of the patches, the unfused areas of the patches filled in and fused subsequently in culture after decanulation to form an intact patch, though the final patch size was smaller with an altered shape. Combining information about the amount of cardiosphere overlap needed (Fig. 4D) with data about cardiosphere dimensions (Fig. 3H,I), we chose to use 33,000 cells per cardiosphere for all subsequent 3D bioprinting experiments, to create cardiospheres between 500 to 550 µm in diameter, that had sufficient overlap for mechanical integrity of the 3D bioprinted cardiac patches. Further assessment of cell viability of the cardiospheres (33,000 cells per cardiosphere, CM:FB:EC ratio 70:15:15) can be found as Supplementary Fig. S4.

### Electrophysiological studies of 3D bioprinted cardiac patches

It has previously been shown that the proportion of cardiomyocytes within a cardiosphere is directly proportional to the dimensional change of the cardiosphere during contraction and the addition of fibroblasts to the cardiospheres improves printed patch integrity^21^. We were interested in the effect of fibroblasts on electrophysiological properties of 3D bioprinted patches, thus we chose three groups of cell ratios to study (CM:FB:EC ratio of 70:0:30, 70:15:15 and 45:40:15).

**Figure 5:**
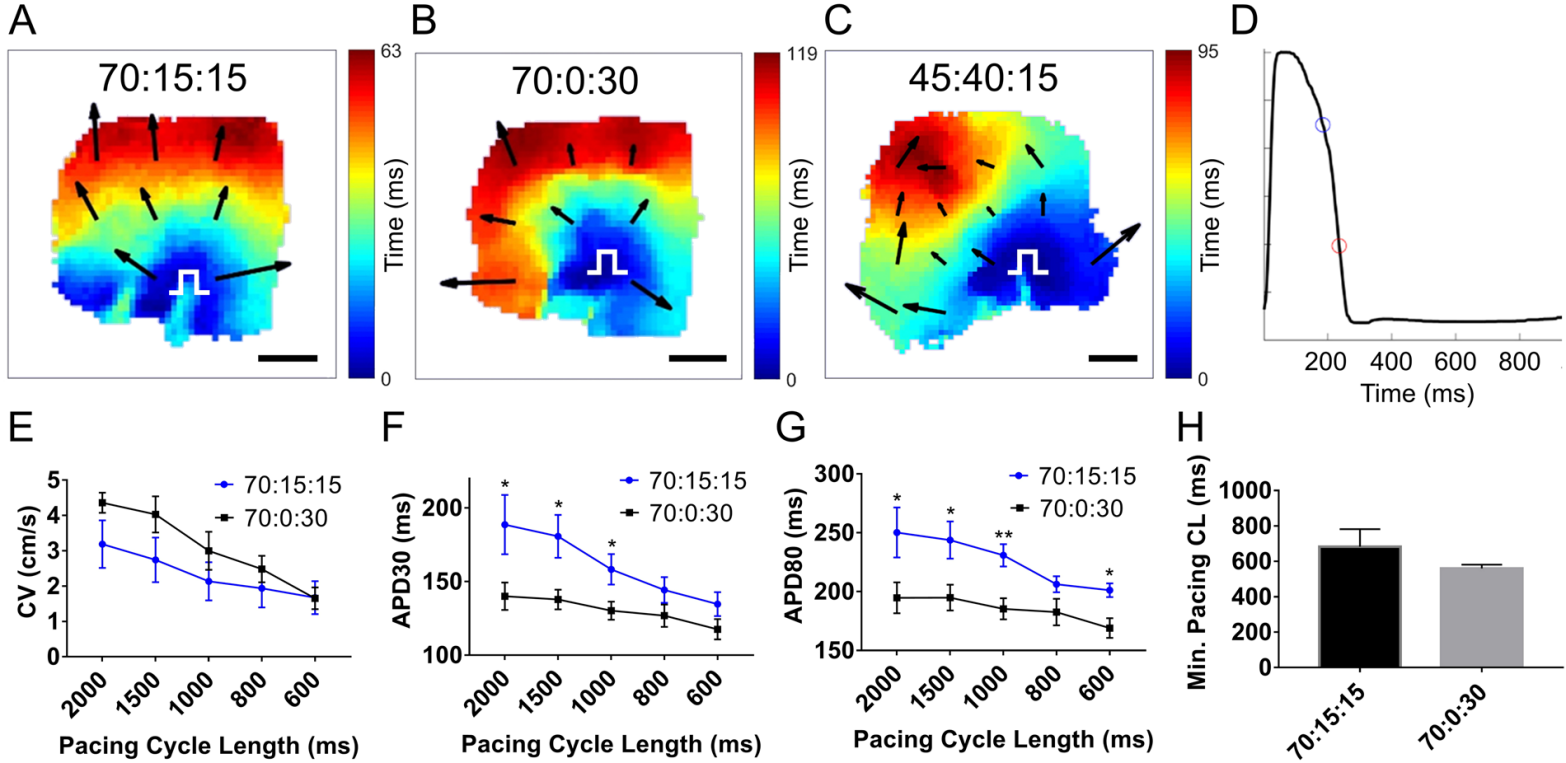
Optical mapping of 3D bioprinted cardiac patches demonstrates electrical integration of component cardiospheres. (**A**–**C**) Representative isochronal activation maps of 3D bioprinted cardiac patches with CM:FB:EC ratios of 70:15:15, 70:0:30 and 45:40:15, respectively. White pulse symbol indicates site of the stimulus electrode. Arrows indicate vector magnitude and direction of activation. Scale bar: 1 mm. (**D**) Representative action potential recording of a 3D bioprinted cardiac patch, with the circles indicating action potential duration at 30% (APD_30_, blue circle) and 80% repolarization (APD_80_, red circle). (**E**–**H**) Mean conduction velocity (**E**), APD_30_ (**F**), APD_80_ (**G**) and minimal pacing cycle length (CL) with 1:1 capture (**H**) of 3D bioprinted cardiac patches (n = 10).

3D bioprinted cardiac patches of CM:FB:EC cell ratios 70:15:15 and 70:0:30 exhibited electrical integration of component cardiospheres one week after bioprinting, could be paced from a point source with electrical capture and propagation across the entire patch, as indicated by isochronal activation maps obtained by voltage optical mapping^22^ (Fig. 5A,B) (n = 10). When a CM:FB:EC cell ratio of 45:40:15 was used, functional block in the middle of the patch, with slower conduction was occasionally observed (Fig. 5C) (n = 3). Conduction velocity (CV) and action potential durations at 30 and 80 percent repolarization (APD_30_, APD_80_)^23^ were calculated for patches (CM:FB:EC 70:0:30 and 70:15:15) at pacing cycle lengths ranging from 2000 to 600 ms (Fig. 5D–G). Patches with CM:FB:EC ratio of 45:40:15 were excluded due to the presence of occasional functional block (Fig. 5C). CV tended to be slower in the 70:15:15 group (n = 6), compared to the 70:0:30 group (n = 4) but this did not reach statistical significance (Fig. 5E). APD_30_ was significantly longer in the 70:15:15 group (n = 6), compared to the 70:0:30 group at the longer pacing cycle lengths (n = 4) (Fig. 5F). APD_80_ was significantly longer in the 70:15:15 group (n = 6), compared to the 70:0:30 group at all pacing rates (n = 4) (Fig. 5G). The minimum pacing cycle length producing capture was not statistically different between the 70:15:15 group and the 70:0:30 group (Fig. 5H).

### Imaging of 3D bioprinted cardiac patches

**Figure 6:**
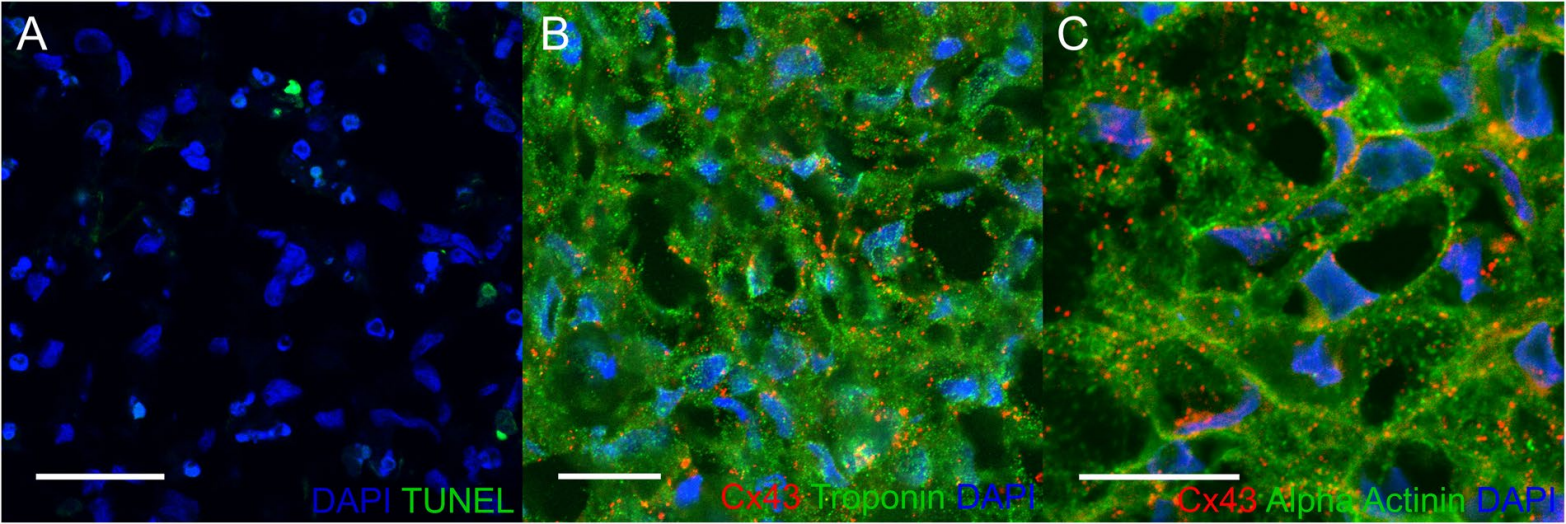
Confocal microscopy of 3D bioprinted cardiac patches. Immunofluorescence of 3D bioprinted cardiac patches (CM:FB:EC 70:15:15) using confocal microscopy. (**A**) TUNEL (Green) DAPI (Blue), Scale bar: 40 µm. Mean patch viability (Live cells/Total cells) = 93.26 ± 5.42% (Mean ± SD). (**B**) Cx43 (Red), Troponin T (Green), DAPI (Blue). Scale bar: 20 µm. (**C**) Cx43 (Red) Alpha-Actinin (Green) DAPI (Blue), Scale bar: 20 µm. Troponin and α-actinin staining demonstrates the development of sarcomeres. Cx43 exhibit punctate staining and clusters of protein at cell contacts.

**Figure 7:**
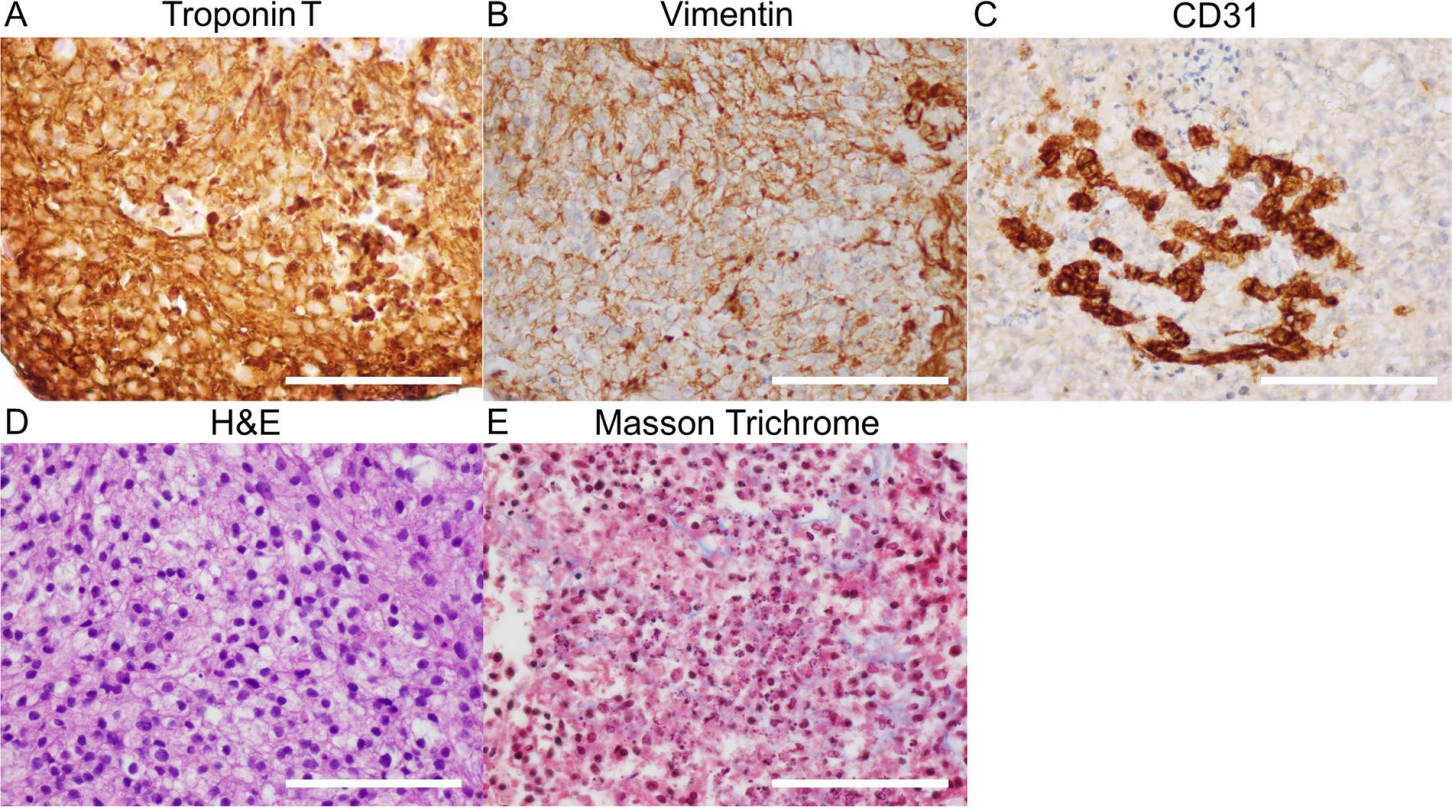
Immunohistochemistry and histology of 3D bioprinted cardiac patches. Tissue staining of 3D bioprinted cardiac patches (CM:FB:EC 70:15:15) with antibodies to Troponin T (**A**), Vimentin (**B**) and CD31 (**C**). (**D**) H&E and (**E**): Masson Trichrome stain. CD31 staining suggests the formation of a rudimentary blood vessel. The tissues are highly cellular and Masson stain reveals the presence of fibrous tissue in the matrix. Scale bar: 100 µm.

The electrophysiological data suggest high cell viability and TUNEL staining demonstrates a mean cell viability of 93.3% one week after bioprinting (Fig. 6A). At this time, the 3D cardiac patches stain positively for markers of each cell type that was incorporated into the cardiospheres: troponin T for CMs, vimentin for FBs and CD31 for ECs (Fig. 7A–C). Interestingly, the pattern of CD31 staining suggests the formation of rudimentary blood vessels (Fig. 7C). Histological stains (H&E and Masson Trichrome) of 3D bioprinted cardiac patches illustrate the presence of collagen indicating the deposition of extracellular matrix after printing of the patch (Fig. 7D,E). Confocal microscopy revealed the presence of connexin 43 (Cx43), a gap junction protein that allows for electrical coupling between cardiomyocytes, localized to cell-cell borders between troponin T (Fig. 6B) and alpha-actinin (Fig. 6C) positive hiPSC-CMs. The distribution of the total number of cells is also comparable between the periphery and the center of the 3D bioprinted cardiac tissue (p = 0.16) (see Supplementary Fig. S5).

### *In vivo* implantation of of 3D bioprinted cardiac patches

**Figure 8:**
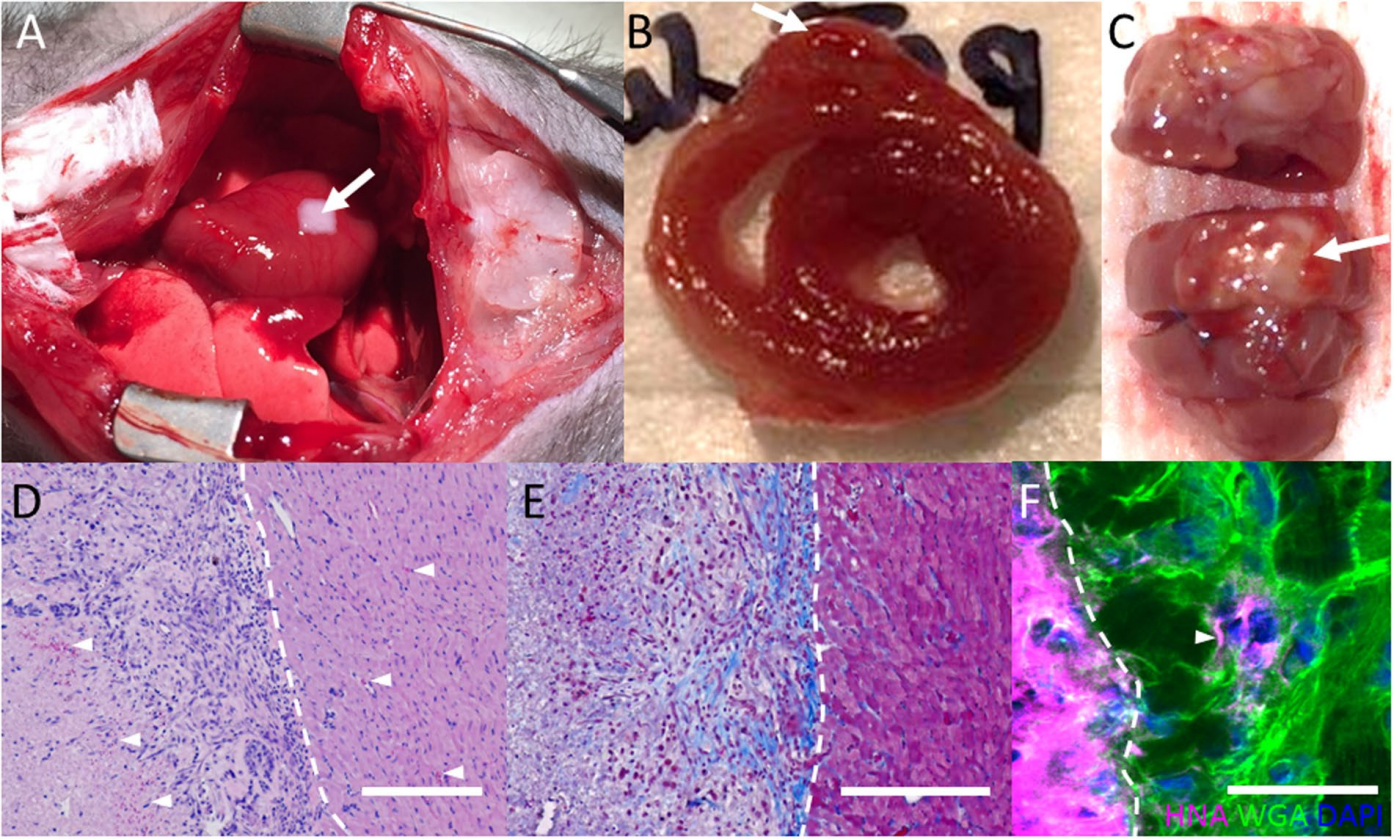
*In vivo* implantation of 3D bioprinted cardiac patches. (**A**) Transplantation of 3D bioprinted cardiac patches (CM:FB:EC 70:15:15) onto the anterior surface of the rat heart. (**B**) Explanted heart (cross-section). (**C**) Explanted heart (anterior aspect). (**D**) H&E, white arrows indicate presence of erythrocytes. Scale bar: 400 µm. (**E**) Masson Trichrome. Scale bar: 400 µm. (**F**) Human Nuclear Antigen (HNA) (Magenta), Wheat Germ Agglutinin (WGA) (Green), DAPI (Blue). Scale bar: 40 µm. White arrows indicate presence of human cells in native rat myocardium. (**D**–**F**) White dotted line demarcates the cardiac patch (left) from native rat myocardium (right).

3D bioprinted cardiac patches were implanted onto nude rat hearts (n = 4) (Fig. 8A–C) and remain engrafted on rat hearts 1 week after implantation (Fig. 8D–F). Imaging of sections through the patch and native heart tissue reveals viable cells in the patch along with erythrocytes (Fig. 8D, left, 3 white arrows), suggesting vascularization. Collagen staining is also present in the patch (Fig. 8E, left). Confocal microscopy reveals presence of human nucleic acid (HNA)-positive cells in native rat myocardium, suggesting engraftment (Fig. 8F, right, white arrow).

## Discussion

Biomaterial-free 3D printing of cardiac tissue avoids the problems associated with using biomaterials^18^ (immunogenicity, fibrous tissue formation, biomaterial degradation, toxicity of degradation products), and also overcomes the difficulties of using cardiosphere self-assembly^21^. It may be difficult to individually place cardiospheres, as well as control the distance between cardiospheres by previously described methods. If cardiospheres are too far apart, tissue voids will be created in the final construct. On the other hand, if cardiospheres are too close together, they may stack and create areas in the patch with increased thickness.

Using our method, we were able to position cardiospheres in precise coordinates, in a flexible grid configuration (Fig. 4A), bioprinting patches of uniform shape and thickness (Figs 4A,B and S2). This results in a more uniform tissue at the macroscopic and microscopic level, compared to previous reported random self-assembly of cardiospheres^21^. Continuous electrical conduction (Fig. 5A,B) also suggests uniform cardiosphere integration.

In the case of cardiosphere self-assembly^21^, it may also be difficult to select individual cardiospheres. Our method allows for the selection of individual cardiospheres, for example, we can instruct the bioprinter to only use cardiospheres between 450 µm to 550 µm in diameter for bioprinting, and reject the use of all others that do not fit this criterion. In addition, 3D bioprinting allows for complex 3D constructs of various geometries (e.g. tubes, rings, etc.) and thicknesses (number of layers) to be created, as well as 3D constructs made of cardiospheres of different cell types, cell ratios and total cell numbers. These complex shapes and structures may be difficult to create using other forms of biomaterial-free cardiac tissue engineering, such as cell sheet technology^24–27^, bioreactor culture systems^28, 29^, including rotating orbital shakers^30, 31^, rapid inter-cell click ligation process^32^ and cardiosphere self-assembly^21^.

In spite of the absence of biomaterials, we were able to achieve high cell density and functional cell contacts, creating a spontaneously beating cardiac tissue. Within 3 days of printing, our 3D bioprinted cardiac patches were spontaneously beating (while on the needle array), and after removal from the array, within another 2 days, the voids in the patch fused and the cardiospheres were electrically integrated (Fig. 5). Due to the absence of exogenous biomaterials in our 3D bioprinted cardiac patches, the local 3D microenvironment may be more physiological^33, 34^, and allow for more cell-cell coupling and communication.

The printed patches reveal a high cell density, a potential drawback is low viability of cells in the bioprinted tissue due to lack of vascuarization. However, we have demonstrated the presence rudimentary blood vessel formed by ECs in the 3D bioprinted cardiac patches (Fig. 7C), and more importantly that the cell viability in the patch is more than 90% (Fig. 6A).

We recognize concerns regarding diffusion limitation, however the 3D bioprinted cardiac patches are histologically and electrically viable, and cell viability measured at a single point in time is high, this does not imply that there is not cell death and turnover. It is possible that several tissue related factors improve oxygen and nutrient diffusion including spontaneous contraction of the patches, the presence of primordial vasculature, less compaction (especially immediately after printing) and prevascularization of the cardiospheres^21^. The cells in the cardiospheres may also exhibit greater tolerance to lower oxygen tension and hypoxic conditioning^35^.

In forming cardiospheres^21, 36^, two important factors that must be considered: (1) the ratio of cell types, (2) cell number. Cardiospheres were not formed when iPSCs or hiPSC-CMs alone were used (Fig. 3A,B); in contrast, wells containing cardiomyocytes co-cultured with at least 15% ECs or FBs formed spheroids (Fig. 3C–I). We kept the EC proportion constant at 15% on the basis of previous reports of successful prevascularization of cardiac patches using ECs as a component^21^. Cardiosphere dimensions depended on cell number and varies slightly with cell composition. (Fig. 3). After optimization, we chose to use 33,000 cells per cardiosphere, to produce cardiospheres that overlap and form patches consistently (Figs 3G,H and 4). We found that in general, an overlap of 50 µm, on each side of the cardiosphere, i.e. cardiosphere diameter of 500 µm, allows for sufficient contact between cardiospheres and for an intact cardiac patch to form 3 days after printing (Fig. 4A).

Fibroblasts may help to stabilize patch structure but these cells are likely to impact myocyte contacts and slow CV. In 3D bioprinted cardiac patches with a higher fibroblast ratio, there is lower CV, and significantly longer APD_30_ and APD_80_ (Fig. 5F,G), suggesting that fibroblasts inhibit electrical coupling of myocardial cells. We also noted the presence of functional block (see Supplementary Video 1) and arrhythmic activity in the middle of the patches with higher fibroblast numbers. A recent study reported similar findings of prolonged APD and increased arrhythmogenicity in *in vitro* cultures of embryonic stem cell-derived cardiomyocytes co-cultured with adult cardiac fibroblasts, compared with co-culture with mesenchymal stem cells^37^. Nonetheless, a non-myocyte cell source, such as fibroblasts, is needed to produce ECM. hiPSC-CMs and ECs will suffice in creating 3D bioprinted cardiac patches, but the slow elaboration of matrix by ECs results in friable patches that cannot be implanted. From a regenerative and biomimicry point of view^38, 39^, the presence of both fibroblasts and endothelial cells is needed, and have been traditionally used by cardiac tissue engineers^40^, attempting myocardial regeneration.

The optical mapping results corroborate the confocal imaging finding, which shows the presence of connexin 43 (Cx43), the main connexin in ventricular myocardium allowing for electrical coupling, expressed between troponin T and alpha-actinin positive hiPSC-CMs (Fig. 6B,C). The absence of other biomaterial in biomaterial-free 3D printing of cardiac patches, may have facilitated the more rapid development of cell-cell contacts containing Cx43 and support more rapid propagation of activation through the patches.

*In vivo* implantations (Fig. 8) suggested vascularization and engraftment of 3D bioprinted cardiac patches, highlighting the promise of biomaterial-free 3D printing of cardiac tissue in tissue regeneration and myocardial repair in the future. Long term follow up and functional analysis of the heart will be needed to show the efficacy of these 3D bioprinted cardiac patches.

The limitations of our study include the short term culture of our 3D bioprinted cardiac patches, the relatively slow conduction velocity of the patches due to immaturity of hiPSC-CMs, the weak mechanical properties of the 3D bioprinted patches. The 3D bioprinted patches consisted of a single layer of spheroid and the properties of a multi-layer thick patch could be different. Notably, the purpose of the *in vivo* experiments was to optimize the conditions for engraftment and vascularization *in vivo*, as a proof of principle. Future studies involving vascularization, different culture conditions and modulation of the tissue with mechanical or electrical stimulation, and implantation in a myocardial injury model, amenable to therapeutics, to assess improvement in function, will be required to more completely assess this technology.

In conclusion, the generation of 3D bioprinted patches created with hiPSC-CMs derived from healthy control subjects is promising as a platform for cardiac tissue repair. The potential to take a patient’s own cells (skin or peripheral blood), reprogram them to hiPSCs then differentiate the hiPSCs to cardiomyocytes is fundamental to the field of regenerative medicine. We have demonstrated it is feasible to 3D print biomaterial-free cardiac tissue patches using hiPSC-CMs. Biomaterial-free 3D printed cardiac tissue created from hiPSCs exhibited spontaneous beating, electrical integration of component cardiospheres, ventricular myocyte-like cellular electrophysiological properties and *in vivo* engraftment and vascularization. This constitutes a significant step towards a new generation of stem cell treatment for heart failure for tissue regeneration, as well as a novel platform for studying heritable cardiac disease mechanisms.

## Methods

### Generation of hiPSC-CMs

Human IPSCs (reprogrammed from the peripheral blood mononuclear cells of a healthy donor) were differentiated into cardiomyocytes, by temporal modulation of Wnt signaling^41^ using small molecules (CHIR99021, Tocris, R&D Systems, Cat. No. 4423, and IWR-1, Sigma-Aldrich, Cat. No. I0161). We only used hiPSC-CMs from regions of 2D cultures that were beating to create cardiospheres. In addition, we also performed optical mapping on the hiPSC-CMs as 2D monolayers for quality control and to ensure that hiPSC-CMs were able to form electrical connections *in vitro* (see Supplementary Fig. S1 and Video 2).

### 3D bioprinting of cardiac patches

We have previously filmed and published our protocol for 3D bioprinting of cardiac patches using only cardiospheres, without the use of biomaterials^19^. In brief, hiPSC-CMs were then co-cultured with human umbilical vein endothelial cells (Lonza, Cat. No. CC-2935) and human adult ventricular cardiac fibroblasts (FB) (Sciencell, Cat. No. 6310) for 72 hours to form mixed cell spheroids (“cardiospheres”) (see Supplementary Fig. S2). A 3D bioprinter (Regenova, Cyfuse Biomedical K.K., Tokyo, Japan) was then used to construct biomaterial-free cardiac patches (Fig. 1). The desired 3D geometry (one single layer of cardiospheres) was prepared using the 3D design software of the 3D bioprinter. The 3D bioprinter first identified the locations of the cardiospheres within U-wells of the ultra-low adhesion 96-well tissue culture plate by automated software detection, a robotic arm then used vacuum suction to pick up, transfer and load the cardiospheres individually onto a needle array in exact spatial coordinates, according to the pre-specified 3D design, in a sterile environment (see Supplementary Fig. S3). Each loading position was calibrated by automated software-aided visual detection of the needle tips to ensure precision and accuracy. Once bioprinted, the cardiac patch was allowed to mature for 72 hours in culture with the needle array in place, while placed on a shaker (Compact Digital Microplate Shaker, Thermo Scientific, Waltham, MA) at 150 rounds per minute (rpm) in the incubator. During this time, the cardiospheres fuse. Then the needle array was removed (decannulation) and the cardiac patches were cultured for variable times before final evaluation and assessment.

### Optical mapping

Cardiac patches were placed in a 35 mm dish and stained with 10 μM of the voltage-sensitive dye di-4-ANEPPS (Sigma-Aldrich Corp., St. Louis, MO) in Tyrode’s solution (135 mM NaCl, 5.4 mM KCl, 1.8 mM CaCl_2_, 1 mM MgCl_2_, 0.33 mM NaH_2_PO_4_, 5 mM HEPES, and 5 mM glucose (Sigma-Aldrich Corp., St. Louis, MO)), supplemented with 20 μM of the contraction inhibitor blebbistatin (Sigma-Aldrich Corp., St. Louis, MO) for 10 minutes at 37 °C. Patches were rinsed twice with Tyrode’s with blebbistatin before being transferred to a stage heated to 37 °C and were allowed to equilibrate for at least 5 minutes before the start of pacing. Samples were point paced with at least 30 stimulus pulses over a range of basic cycle lengths, starting from 2000 ms and decrementing until loss of capture. Optical recordings during pacing were obtained using a 100 × 100 pixel CMOS camera (MiCAM Ultima-L, SciMedia, Costa Mesa, CA).

Optical mapping data was analyzed using custom MATLAB scripts. Recordings at each pixel were de-noised using a previously described method^42^ to regulate total signal variance and convolved with a 5 × 5 spatial Gaussian filter. Activation times, defined as the maximum of the derivative of membrane potential (dV/dt), were calculated as previously described^43^. Histograms of local conduction velocities were generated for each patch and were fitted to a Gaussian curve. The mean of the curve was defined as the average conduction velocity (CV). For each patch, action potential durations at 30 and 80 percent repolarization (APD_30_ and APD_80_) were calculated for all local traces over the recording area and fit with Gaussian curves to determine the mean value for each sample, as described for CV measurements.

### Flow Cytometry

Flow cytometry was performed on single cells dissociated from 2D hiPSC-CM monolayers using a flow cytometer (Accuri C6 Flow Cytometer, BD Biosciences, San Jose, CA) using the antibodies VCAM1, VEGFR2, PDGFRβ (BD Biosciences, San Jose, CA).

### Immunofluorescence

Printed tissues were fixed in 4% paraformaldehyde, embedded in O.C.T. compound (Tissue-Plus™, Thermo Fisher, Waltham, MA), and cut into 8 µm sections. The tissues were then washed in PBS, incubated in 0.5% Triton X for 5–10 minutes, washed in PBS again and blocked for one hour using 10% goat serum. Primary antibodies to the cell protein or biomarker of interest (Troponin, Alpha-Actinin, Connexin 43 (Cx43), Human Nucleic Acid (HNA), Wheat Germ Agglutinin (WGA)) were incubated with the sections overnight. Removal of unbound primary antibodies was accomplished by washing using PBS. Secondary antibodies were incubated with the sections for one hour before washing using PBS and mounting using Vectashield Antifade Mounting Medium with 4′,6-diamidino-2-phenylindole (DAPI) (Vector Laboratories, Burlingame, CA).

### Immunohistochemistry

High-temperature antigen retrieval and paraffin removal were performed by immersing slides in Trilogy (Cell Marque, Hot Springs, AR) in a pressure cooker until the chamber reached 126 °C and 23 psi. A Dual Endogenous block (Dako, Carpinteria, CA) was applied for 5 minutes. Slides were incubated with antibodies to Vimentin (Cell Signaling Technology, Danvers, MA), cardiac Troponin T (Abcam, Cambridge, MA), or CD31 (Thermo Fisher, Waltham, MA), followed by addition of a mouse or rabbit HRP detection system (Leica Biosystems, Chicago, IL). Finally, slides were stained with a DAB substrate (Vector Lab, Burlingame, CA) and counterstained with hematoxylin (Richard-Allen Scientific, Kalamazoo, MI).

### Cell Viability Assay

Cell viability of the 3D bioprinted cardiac tissue was determined using TUNEL assay (DeadEnd™ Fluorometric TUNEL System, Promega, Madison, WI) on 3D bioprinted cardiac tissue that was removed from cell culture media and immediately embedded in O.C.T. compound (Tissue-Plus™, Thermo Fisher, Waltham, MA), then cross-sectioned into 8 µm sections and stained. Live and dead cell counts were counted using ImageJ software on representative images in the periphery (n = 4) and the center (n = 4) of the 3D bioprinted cardiac tissue.

Cell viability of the cardiospheres were determined using a kit that marks live cells (the presence of ubiquitous intracellular esterase activity enzymatically converts nonfluorescent cell-permeant calcein AM to green fluorescent calcein) and dead cells with (ethidium homodimer enters dead cells with damaged membranes and binding to nucleic acids with red fluorescence) (LIVE/DEAD® Viability/Cytotoxicity Kit for mammalian cells #L3224, Thermo Fisher, Waltham, MA). Imaging of cardiospheres was then performed using a confocal laser scanning microscope (Zeiss LSM800 with upgraded GaAsP detectors).

Cell viability of the cardiospheres was also measured using a 3D cell viability assay (CellTiter-Glo #G9681, Promega, Madison, WI). Briefly, the spheroids/cells were mixed vigorously with the CellTiter-Glo 3D reagent for 5 minutes and incubated at room temperature for 25 minutes to stabilize luminescence. It was then read by a plate reader (Molecular Devices Spectra MAX Gemini EM).

### Imaging

Photographs of 3D bioprinted cardiac tissue and the 3D bioprinting process were acquired using a 8 MP digital camera. 3D bioprinted cardiac patches were imaged using inverted microscopes (EVOS XL, EVOS FL Cell Imaging System, Thermo Fisher, Waltham, MA). Confocal microscopy was performed using an upright Zeiss LSM 700 or LSM 510 Meta with oil-immersion objectives ranging from 40× to 63× using spectral lasers at 405-, 488- and 561-nm wavelengths. ImageJ software (NIH, Bethesda, MD) was used to generate composite microscopy images by combining fluorescent channels.

### *In vivo* implantation

Animal procedures were reviewed and approved by the Johns Hopkins University Institutional Animal Care and Use Committee and performed in accordance with relevant institutional and federal guidelines and regulations for the care and use of laboratory animals. Nude rats (NIH RNU sp/sp rats, NTac:NIH-*Foxn1* ^*rnu*^, 12 weeks, female, 165–195 g, Taconic) were anesthetized using isoflurane, intubated and mechanically ventilated. A median sternotomy was performed and hemostasis was secured. A single cardiac patch was implanted directly onto the heart, and secured with tissue glue and sutures as needed. The chest was closed under aseptic technique and the animals were monitored for recovery from surgery. Animals were sacrificed 1 week after patch implantation and their hearts were explanted. Hearts were sectioned along the short axis and embedded in O.C.T. compound or paraffin, for further histology and immunostaining.

### Statistics

For all experiments, data was represented graphically as bar or line charts with error bars, representing the mean with the standard error of the mean, using Prism 7 (GraphPad, La Jolla, CA). Statistical analysis of CV, APD, minimum pacing cycle length and cell viability was performed with unpaired 2-tailed t tests. A p value less than 0.05 was considered statistically significant.

### Data availability statement

All data generated or analysed during this study are included in this published article (and its Supplementary Information files).

## Electronic supplementary material

Supplementary Information

Video 1

Video 2
