# Supplementary material for: Biomaterial-Free Three-Dimensional Bioprinting of Cardiac Tissue using Human Induced Pluripotent Stem Cell Derived Cardiomyocytes
Source: Sci Rep. 2017 Jul 4;7:4566. doi: 10.1038/s41598-017-05018-4 (PMC5496874; doi:10.1038/s41598-017-05018-4)
Supplement: Supplementary file 1 — Supplementary Information [file 41598_2017_5018_MOESM1_ESM.pdf]

Supplementary Information

Biomaterial-Free Three-Dimensional Bioprinting of Cardiac Tissue using Human Induced Pluripotent Stem Cell Derived Cardiomyocytes

Chin Siang Ong, MBBS[1,2], Takuma Fukunishi, MD[1], Huaitao Zhang, BS[1], Chen Yu Huang, PhD[2], Andrew Nashed, MD[2], Adriana Blazeski, MS[3], Deborah DiSilvestre, MS[2], Luca Vricella, MD[1], John Conte, MD[1], Leslie Tung, PhD[3], Gordon F. Tomaselli, MD[2], Narutoshi Hibino, MD PhD[1*]

[1]Division of Cardiac Surgery, Johns Hopkins Hospital, Baltimore, Maryland, USA
[2]Division of Cardiology, Johns Hopkins Hospital, Baltimore, Maryland, USA
[3]Department of Biomedical Engineering, Johns Hopkins University, Baltimore, MD, USA

*Corresponding Author:
Narutoshi Hibino, MD, PhD
Division of Cardiac Surgery
The Johns Hopkins Hospital
Zayed 7107, 1800 Orleans St, Baltimore, MD 21287, USA
Phone number: +1(410)502-7683
Email: nhibino1@jhmi.edu

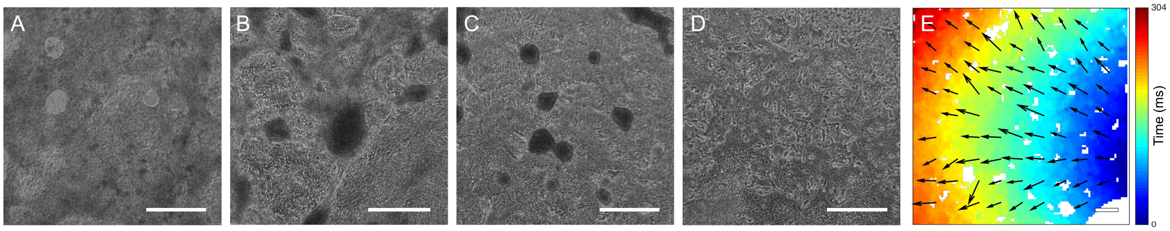

Figure S1: hiPSC-CM 2D monolayer quality by spontaneous beating phenotype and optical electrical mapping.

A-D : Optical microscopy of 2D CM monolayer as a connected beating sheet (A), with connected beating areas (B), isolated beating areas (C) and that are not beating (D). Scale bar: 500 µm. E: Isochronal activation map of hiPSC-CM 2D monolayer by optical mapping using a voltage sensitive dye. Scale bar: 1 mm.

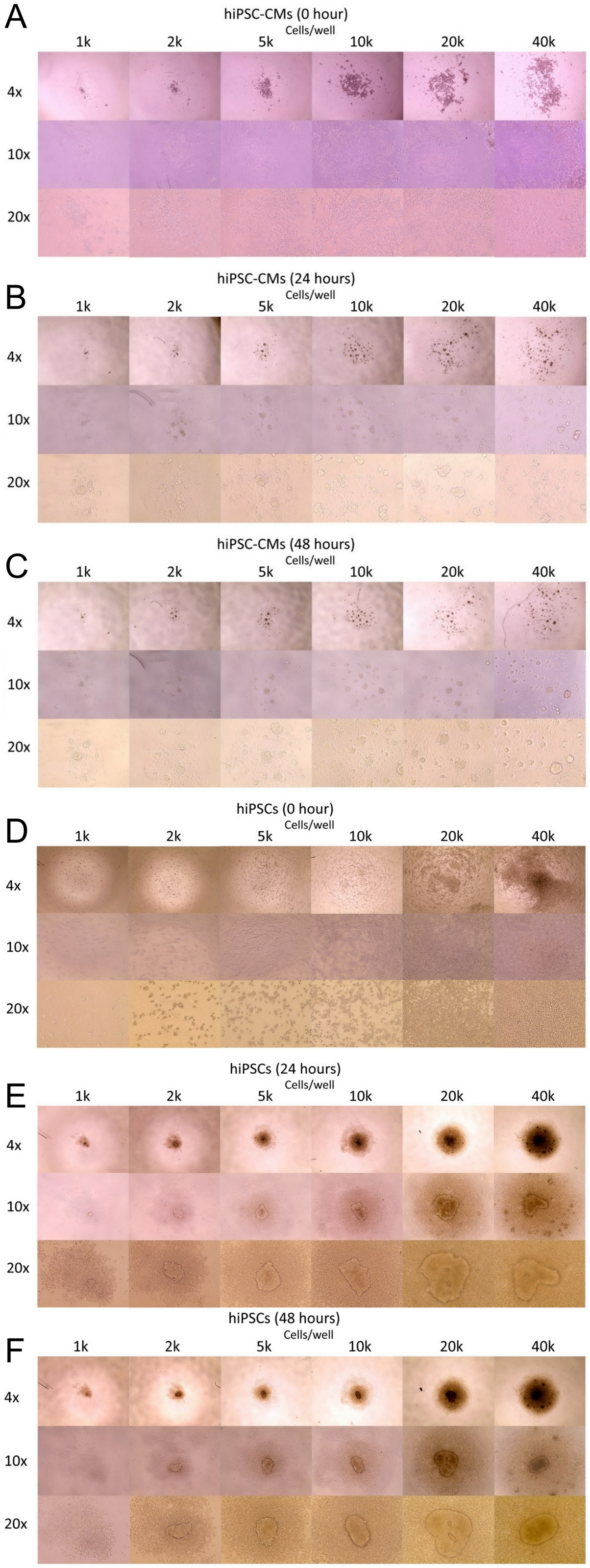

Figure S2: Cardiosphere optimization studies.

A-C: IPSCs (100%) do not form spheroids after 24 hours (B) and 48 hours (C) in ultra-low attachment 96-well plates (Top row: 4x magnification, Middle row: 10x magnification Bottom row: 20x magnification). Instead a soft gel-like cell aggregate, that is easily disrupted, forms within 24 hours.

D-F: hiPSC-CMs (100%) do not form spheroids after 24 hours (B) and 48 hours (C) in ultra-low attachment 96-well plates (Top row: 4x magnification, Middle row: 10x magnification Bottom row: 20x magnification). Instead, multiple small scattered spheroids form within 24 hours, which persists at 48 hours.

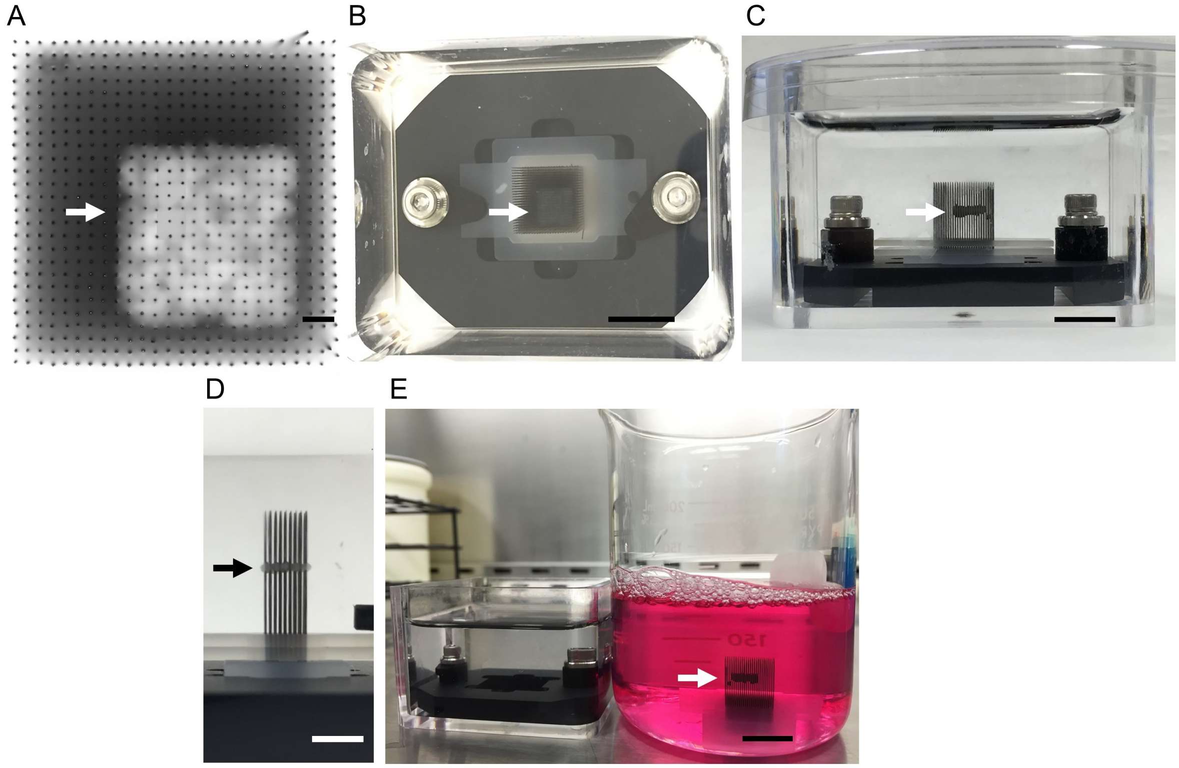

Figure S3: Additional images of 3D bioprinted cardiac patches.

A: Top view of a 3D bioprinted cardiac patch (white arrow) on a 26 x 26 needle array. Scale bar: 1 mm.

B: Top view of a 3D bioprinted cardiac patch (white arrow) on a 26 x 26 needle array. Scale bar: 10 mm.

C: Side view of a 3D bioprinted cardiac patch (white arrow) on a 26 x 26 needle array. Scale bar: 10 mm.

D: Side view of a 3D bioprinted cardiac patch (black arrow) on a 9 x 9 needle array. Scale bar: 4 mm.

E: Side view of a 3D bioprinted cardiac patch (white arrow) on a 26 x 26 needle array after being transferred into a beaker with cell culture media. Scale bar: 10 mm.

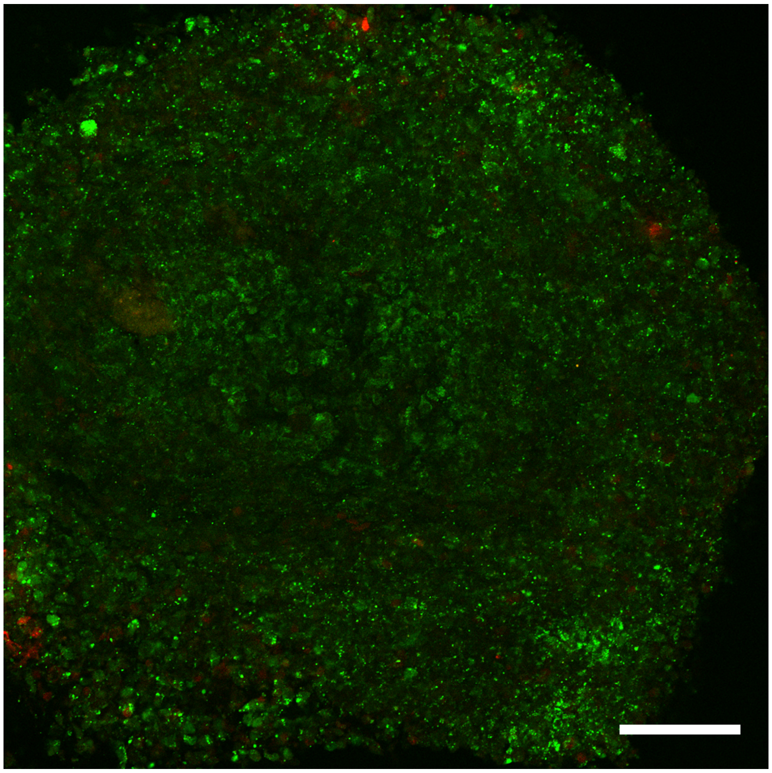

Figure S4: Cardiosphere cell viability.
Cell viability assessment by confocal imaging of the mid-section of a cardiosphere stained with cytotoxicity kit (live cells green, dead cells red). Scale bar: 100 µm

Note: The cell viability of the cardiospheres (33,000 cells per cardiosphere, CM:FB:EC ratio 70:15:15) assessed by 3D cell viability ATP assay was 93.3%. The standard curve was generated by using cell mixtures consisting of 10^4, 10^5, 10^6 cells (CM:FB:EC 70:15:15).

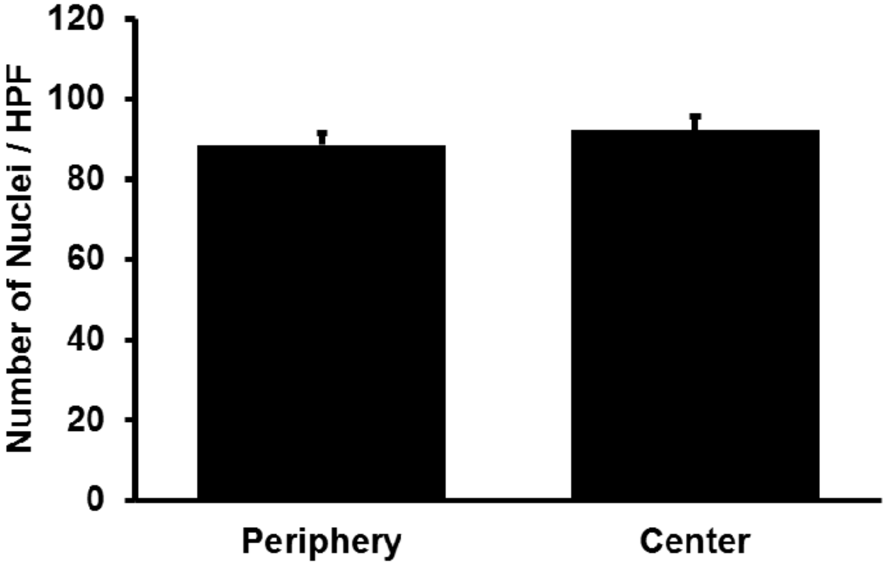

Figure S5: Cell distribution in 3D bioprinted cardiac tissue.
Number of nuclei per HPF (40x) of 3D bioprinted cardiac patch in the periphery: 88.5 ± 3.1 nuclei/HPF (n=4) vs. center: 92.3 ± 3.5 nuclei/HPF (n=4) (Number of nuclei/HPF ± SD) (p = 0.16).

Video 1.mov

Uploaded as a separate file

Video 1: Representative optical mapping video of 3D bioprinted cardiac patch (CM:FB:EC 45:40:15). Top: 3D bioprinted cardiac patch. Middle: Action potential recording at the magenta dot in the 3D bioprinted cardiac patch. Bottom: Pacing stimulus (Pacing cycle length 2000 ms).

Video 2.mov

Uploaded as a separate file

Video 2: Video microscopy of 2D CM monolayers.
Scale bar: 500 µm. (Video version of Figures 3E-H.)
A: Connected beating sheet (Grade A).
B: Connected beating areas (Grade B).
C: Isolated beating areas (Grade C).
D: Not beating (Grade D).
